# Comparison of Lower Limb Joint Reaction Forces in Patients with Cerebral Palsy and Typically Developing Individuals

**DOI:** 10.3390/medicina61020246

**Published:** 2025-01-31

**Authors:** Yasar Mahsut Dincel, Alina Nawab Kidwai, Kerim Atmaca, Nese Aral Sozener, Yunus Ziya Arslan

**Affiliations:** 1 Department of Orthopedics and Traumatology, Faculty of Medicine, Namık Kemal University, 59030 Tekirdag, Türkiye; ymdincel@nku.edu.tr; 2 Department of Mechanical Engineering, Faculty of Engineering, Middle East Technical University, 06800 Ankara, Türkiye; alina.kidwai@metu.edu.tr; 3 Department of Mechanical Engineering, Faculty of Engineering, Turkish-German University, 34820 Istanbul, Türkiye; kerim.atmaca@tau.edu.tr; 4 Department of Molecular Biotechnology, Faculty of Science, Turkish-German University, 34820 Istanbul, Türkiye; aral@tau.edu.tr; 5 Department of Robotics and Intelligent Systems, Institute of Graduate Studies in Science and Engineering, Turkish-German University, 34820 Istanbul, Türkiye

**Keywords:** joint reaction force, cerebral palsy, joint kinetics, OpenSim, comparative musculoskeletal simulation

## Abstract

*Background and Objectives*: Kinematic and kinetic data from gait analysis are commonly used for clinical decision making in cerebral palsy (CP). However, these data may not fully capture the underlying causes of movement pathologies or effectively monitor post-treatment changes. Joint reaction forces (JRFs), estimated through simulation-based methods, provide valuable insights into the functional state of musculoskeletal components. Despite their importance, comprehensive evaluations of lower limb JRFs in CP are limited, and comparisons with typically developing (TD) individuals remain underexplored. This study aimed to provide a detailed comparison of lower limb JRFs between children with CP exhibiting mild crouch gait and age-matched TD children during self-selected walking speeds. *Materials and Methods*: Open-access gait datasets from eight children with CP and eight TD children were analyzed. A full-body musculoskeletal model was scaled to individual anthropometric data in OpenSim. Joint angles and moments were obtained using inverse kinematics and inverse dynamics, respectively. Ankle, knee, and hip JRFs were calculated using OpenSim’s Joint Reaction tool. Root-mean-square differences and Pearson correlation coefficients quantified the differences between CP and TD JRFs. *Results*: The anterior–posterior and vertical components of the hip JRFs in CP were lower than in TD children. CP knee JRFs exceeded TD values across all anatomical axes. For the ankle, the anterior–posterior JRF was lower in CP, whereas the vertical component was higher compared to TD. *Conclusions*: Children with CP experience distinct lower limb JRF patterns compared to TD children. While some findings align with previous studies, discrepancies in other components highlight the influence of model and patient-specific characteristics. These results emphasize the need for standardization in reporting patient data and systematic evaluations to improve the interpretation and applicability of JRF analyses in CP research and treatment planning.

## 1. Introduction

Joint reaction force (JRF) is generated at the joint in response to the external loads and muscular forces during movement. In the case of cerebral palsy (CP), JRFs in the ankle, knee, and hip joints can potentially play an essential role in treatment. These forces provide critical information about the structural loading patterns during gait and other activities, which can help clinicians tailor treatment plans and monitor progress [1]. Abnormal JRFs have been deemed a cause of bony deformities in children with CP, impacting bone growth [2]. The investigation of JRFs can enable the determination of the contribution of different muscle groups to joint load [3,4]. For example, elevated quadriceps forces were found to be a major contributor to abnormal compressive tibiofemoral force in patients with CP [5]. The utility of JRFs, however, is not limited to aiding the understanding of pathomechanics. Interventions producing similar improvements in kinematics can be compared in terms of JRFs. For example, in a study comparing botulinum toxin injections and single-event multilevel surgery, only the latter effectively improved joint loading in CP despite both interventions leading to significant enhancements in lower limb joint kinematics [6]. The accurate estimation of JRFs can also shed light on the design of orthopedic implants [7] as well as on assessing the risk of injury [8].

JRFs have been investigated extensively in children with CP. Tibiofemoral contact forces have been found to be as high as six times the subject’s body weight during severely crouched walking in CP [5,9], with patellofemoral contact forces nearly five times the body weight [9] and hip JRFs elevated [10], exemplifying the debilitating nature of the pathology. Abnormal kinematics and bony deformity can both influence JRFs in CP, with JRF and finite element analysis studies suggesting that the former may induce the latter [2,10,11]. Further, a recent study investigating skeletal deformity development in CP found hip JRF orientation in the sagittal plane to be the main determiner of simulated femoral growth, where it was also found that CP patients without femoral deformities had more normal hip JRFs due to unique frontal plane hip kinematics reducing the detrimental effects of abnormal sagittal plane kinematics on hip JRF—a quality that differentiated them from CP patients with femoral deformities [12]. This exemplification of kinematic strategies and their effect on joint load and bone remodeling presents strong support for the utility of JRF analyses in clinical practice for CP, given that similar investigations for knee osteoarthritis have seen a clinical impact [13]. Apart from kinematic strategies, invasive interventions can also be assessed in terms of induced joint load normalization. In patients with CP, JRFs in all lower limb joints showed improvement following single-event multilevel surgery, whereas improvements were limited to the ankle joint after botulinum toxin injections (BTI) [6,14].

Several approaches exist to measure joint loading. In vivo measurements are often impractical due to their invasive nature, which can alter natural joint function and raise ethical concerns. Most joint loading data are typically derived from instrumented implants, which may not accurately reflect the reaction forces in intact joints [15]. Consequently, model-based estimations serve as a safe and practical alternative. JRFs can be computed using various optimization algorithms within musculoskeletal simulations, which predict muscle forces based on inverse dynamics analysis by incorporating joint kinematics and ground reaction force data [16,17]. Since solving the equations of motion alone does not yield internal forces, the standard approach involves recursively solving the Newton–Euler equations. This process uses muscle forces and joint kinematics, beginning with the foot segment in contact with the external ground reaction force, and continues up to the joint of interest [5,18]. The resulting JRF includes the load transmitted through the unmodeled joint structures (e.g., cartilage, ligaments) under the specified kinematic conditions [5].

The physiological implications of differences in JRFs between patients with CP and typically developing (TD) populations would be of clinical relevance. To enhance our understanding of joint kinetics in patients with CP, this study aimed to investigate the differences in JRFs at the hip, knee, and ankle joints between patients with CP and TD individuals.

## 2. Materials and Methods

### 2.1. Dataset

The open-access gait data of eight patients with CP and mild crouch gait (age: 8.3 ± 1.6 yrs, height: 1.21 ± 0.11 m, mass: 26.9 ± 9.2 kg) made available by Steele et al. [19] were employed in our study. The dataset consisted of marker-based kinematic data obtained using a 12-camera motion capture system (Vicon Motion Systems, Lake Forest, CA, USA) with a standard thirteen-marker protocol and ground reaction forces from four force platforms (AMTI, Watertown, MA, USA) recorded while subjects walked at self-selected speeds (0.91 ± 0.14 m/s). The inclusion criteria were (i) a diagnosis of diplegic CP with mild crouch gait (minimum knee flexion angle during the stance phase between 15° and 40°); (ii) the ability to achieve at least 0° of ankle dorsiflexion during a physical exam, with no equinus during walking; (iii) no history of prior surgeries; and (iv) tibial torsion and femoral anteversion angles below 30°. Additionally, open-access gait data from eight age-matched TD individuals (age: 9.5 ± 2.1 years, height: 1.28 ± 0.19 m, mass: 31.5 ± 7.9 kg) were used to obtain normative gait patterns [20].

### 2.2. Musculoskeletal Modeling and Simulation

The Rajagopal full-body musculoskeletal model was used, comprising 37 degrees of freedom, 22 rigid body segments, and 80 Hill-type muscle–tendon actuators [21], and is available in OpenSim [17]. The muscle–tendon unit parameters in the model are based on data from healthy adults [22] and cadaveric studies [23]. The generic musculoskeletal model was scaled to match each individual’s anthropometric measurements by minimizing the difference between virtual marker positions and experimentally recorded marker positions in a static pose.

**Figure 1:**
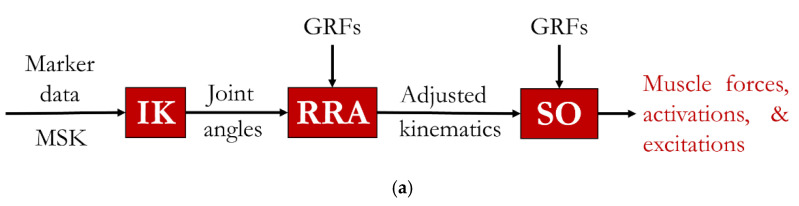

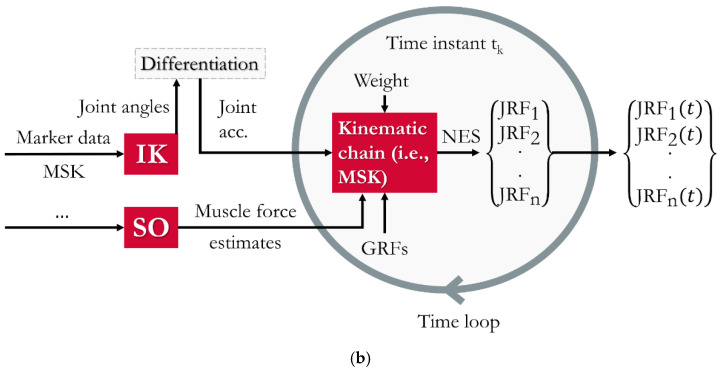
(**a**) Muscle force estimation pipeline. MSK: scaled musculoskeletal model; IK: inverse kinematics; RRA: residual reduction algorithm; GRFs: ground reaction forces; SO: static optimization. (**b**) OpenSim Joint Reaction Analysis tool. acc.: accelerations; NES: Newton–Euler solution; JRFn: joint reaction force of the nth joint; JRFn(*t*): joint reaction force profile of the nth joint over the gait cycle.

Joint angles during walking were calculated using inverse kinematics, with a maximum marker error of less than 2 cm. These joint angles, along with ground reaction force data, were input into the static optimization tool to compute muscle forces (Figure 1a). JRFs were subsequently estimated using the Joint Reaction Analysis tool in OpenSim (Figure 1b).

In OpenSim, the musculoskeletal model is represented as a kinematic chain, and a force balance is calculated for each time point of the gait cycle. Equation (1) illustrates the vector sums used in this analysis [5], computed in the reference frame of the segment immediately distal to the joint:

(1) $${\vec{R}}_{joint}=\left[\begin{array}{c} {\vec{\tau }}_{joint}\\ {\vec{F}}_{joint} \end{array}\right]={\left[M\right]}_{ds}{\vec{a}}_{ds}-({\vec{R}}_{pj}+\sum {\vec{F}}_{muscles}+{\vec{F}}_{gravity})$$

where $\vec{R}$*_joint_* is the generalized force vector containing the JRF ($\vec{F}$*_joint_*) and joint reaction moment ($\vec{\tau }$*_joint_*) of the joint of interest, [*M*]*_ds_* is the generalized mass matrix of the segment distal to the joint, $\vec{a}$*_ds_* describes the acceleration of this segment, $\vec{R}$*_pj_* is the generalized force vector of the previous (distal) joint, $\sum \vec{F}$*_muscles_* is the sum of the muscle forces on the segment distal to the joint of interest, and $\vec{F}$*_gravity_* describes the force of gravity on this distal segment. Initially, the JRFs and joint reaction moments are computed in the reference frame of the segment distal to the joint of interest (specifically at the segment origin); however, OpenSim ultimately expresses these forces and moments in the joint center frame.

### 2.3. Data Analysis

Pearson correlation coefficient (PCC) and root-mean-squared difference (RMSD) values between the average of normative JRFs obtained from TD individuals and the JRFs of each CP patient were calculated and statistically analyzed in SPSS (Version 21.0; SPSS; Chicago, IL, USA), with the level of statistical significance set to 0.05 (*p* < 0.05). The Shapiro–Wilk test confirmed that the data were not normally distributed. Therefore, a non-parametric Mann–Whitney U test was performed. A Bonferroni correction was applied to adjust the *p*-value for multiple comparisons (*p* < 0.016).

## 3. Results

**Figure 2:**
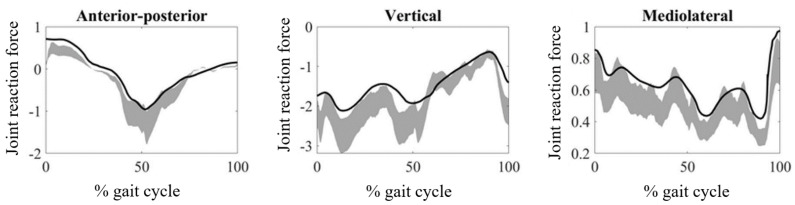
Hip joint reaction forces during walking obtained from CP patients. The gray zones indicate normative hip joint reaction forces from typically developing individuals.

**Figure 3:**
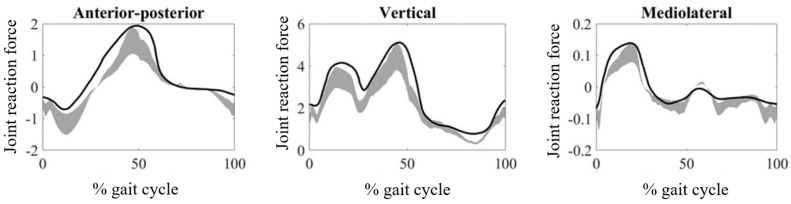
Knee joint reaction forces during walking obtained from CP patients. The gray zones indicate normative knee joint reaction forces from typically developing individuals.

**Figure 4:**
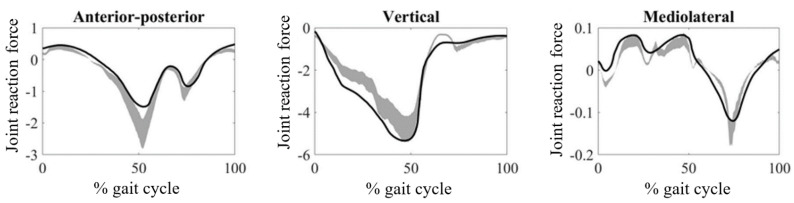
Ankle joint reaction forces during walking obtained from CP patients. The gray zones indicate normative ankle joint reaction forces from typically developing individuals.

The JRFs obtained from the hip, knee, and ankle joints are provided in Figure 2, Figure 3 and Figure 4, respectively. The magnitudes of the JRFs were normalized to the body weight (BW) of each subject.

For the hip joint, the anterior–posterior and vertical components of the JRF were lower than those of TD individuals, while the mediolateral component was higher than normative data (Figure 2). In terms of mean RMSD values, the maximum mean RMSD (0.41) was obtained from the mediolateral component, while the minimum (0.15) was obtained from the anterior–posterior component (Table 1). The RMSD value for the anterior–posterior component was found to be significantly different to those obtained for the vertical (*p* = 0.012) and mediolateral components (*p* = 0.012). In terms of mean PCC values, the maximum mean PCC value (0.91) was obtained for the anterior–posterior component, while the minimum (0.83) was obtained for the vertical component (Table 2). The PCC value for the anterior–posterior component was found to be significantly different to those obtained for the vertical (*p* = 0.013) and mediolateral components (*p* = 0.014).

In the knee joint, JRFs of CP patients in all anatomical axes were higher than those obtained for TD individuals (Figure 3). In terms of mean RMSD values, the maximum mean RMSD value (0.28) was obtained from the vertical component, while the minimum (0.22) was obtained from the mediolateral component (Table 1). No significant differences between the RMSD values of the three components of JRF were found (*p* > 0.016). In terms of mean PCC values, the maximum mean PCC (0.90) was obtained for the anterior–posterior component, while the minimum (0.87) was obtained for the mediolateral component (Table 2). The only significant difference was found between the PCC values obtained for the anterior–posterior and mediolateral components (*p* = 0.014).

In the ankle joint, the JRF along the anterior–posterior axis is lower in CP patients than in the TD population and vice versa along the vertical axis (Figure 4). In terms of mean RMSD values, the maximum mean RMSD value (0.32) was obtained from the vertical component, while the minimum (0.26) was obtained from the anterior–posterior component (Table 1). There was no significant difference between the RMSD values of the components (*p* > 0.016). In terms of mean PCC values, the maximum mean PCC value (0.84) was obtained from the vertical component, while the minimum (0.79) was obtained from the mediolateral component (Table 2). The PCC value of the vertical component was found to be significantly different to those obtained for anterior–posterior (*p* = 0.014) and mediolateral components (*p* = 0.014).

## 4. Discussion

This study aimed to compare the hip, knee, and ankle JRFs of patients with CP exhibiting a mildly crouching gait to those of TD children. At the hip, the JRF in CP patients was oriented more anteriorly, medially, and superiorly compared to TD individuals. Similar findings were observed at the knee. In contrast, at the ankle, the JRF in CP patients was more inferiorly oriented.

Recently, joint kinetics, especially joint moments and joint forces, have become an additional tool in evaluating normal and pathological gait [24,25]. JRFs can play an integral role in the comprehensive assessment of individuals with CP, with the potential to assist in the identification of primary causes of movement pathology and guide surgeons in making informed decisions about the type, timing, and extent of surgical interventions. A patient-specific approach helps optimize functional outcomes and addresses the unique musculoskeletal challenges associated with CP [26]. Furthermore, after surgery, monitoring JRFs can help evaluate the effectiveness of the surgical intervention. Assessing how joint forces change postoperatively can aid in determining if the surgical goals, such as improved joint alignment and reduced deformity, have been achieved sustainably, specifically by evaluating the level of joint load normalization.

Several studies have investigated JRFs in the CP population. However, due to the heterogeneity of patient characteristics such as joint kinematics, gait pattern, and the type of CP, as well as method of presenting JRF results, only three studies [10,12,27] were found suitable for comparison with the results of our study. To ensure consistency, results from generic models were considered where both generic and personalized models were available. While all the subjects of the study by Kainz and Jonkers [27] had diplegic CP and mild crouch, eligible subject subsets were chosen from the remaining studies. Carriero et al. [10] investigated only the hip, Kainz et al. [12] investigated the hip and knee, and Kainz and Jonkers [27] investigated the hip, knee, and ankle.

The comparison between our findings and those of previous studies reveals both similarities and differences. Across all studies, the hip JRFs predominantly exhibited posterior and inferior orientations; however, our study demonstrated fewer undulations in these components over time. Notably, Carriero et al. [10] found considerable differences in the hip JRF profiles of children with CP compared to typical controls, possibly due to variations in modeled bone morphologies and patient characteristics, such as a higher prevalence of stiff knee gait. Contrastingly, the remaining studies, including the present, showed much similarity in hip JRF profile shape between TD and CP subjects. In terms of the mediolateral component of hip JRF, our findings contrast with previous studies, as we observe a consistent medial orientation throughout the gait cycle. This difference can potentially be attributed to the utilization of the Rajagopal model with a higher number of model segments and degrees of freedom, as these factors have been shown to affect muscle force estimates [28], and can thus affect JRFs as well. Across all studies, the anteroposterior component of hip JRF in CP patients is consistently lower than TD counterparts, with Carriero et al. [10] reporting the lowest magnitudes.

For the knee joint, our results for the mediolateral component are quite similar in profile but often lower in magnitude compared to Kainz et al. [12] and Kainz and Jonkers [27]. Notable differences are found for the remaining components. The anterior–posterior knee JRF pattern of the present study displays a single peak around the time of swing, but a double peak pattern is seen in the other studies, with peaks occurring during loading response and terminal stance. The vertical component of knee JRF displays the double hump profile presented by the other studies. However, it is superiorly oriented rather than inferiorly. These differences can again be attributed to the use of the Rajagopal model in the present study, which has a greater number of segments, muscles, and degrees of freedom than the other studies.

Regarding ankle JRF, our results closely resemble those of Kainz and Jonkers [27], albeit with some differences in the anteroposterior component, possibly due to variations in muscle activation patterns. The consistent medial orientation of the ankle mediolateral JRF in our study may reflect the specific characteristics of our patient group, but model effects may be a likelier cause.

The commonly observed weakness of plantar flexor muscles (that are important for forward propulsion [29]) in CP, as well as weak hip extensors [30], may be among the causes of differences between CP and TD JRFs in our study. Hip extensors have been shown to cause an anteriorly acting hip contact force component [4], and the greater than normal anterior hip JRF during early stance in our study may thus be reflective of hip extensor weakness. In contrast to Yadav et al. [4], Correa et al. [3] found iliopsoas force to be a major contributor to anterior hip JRF. This may indicate a need to consider results from different techniques alongside measurable variables to enable a holistic understanding of muscle contributions to JRFs. For example, using muscle strength assessments in conjunction with JRFs can reveal important insights into the contribution of muscle weakness to JRF, given that ankle plantar flexor moment has been found to correlate with the strength of six lower extremity muscle groups [31]. The crouched posture, which requires higher forces [5] to be generated by muscles to stabilize and control the knee, is another factor in the departure of CP JRFs from normal.

Several limitations of this study should be noted. First, the generic Rajagopal model used in this study defines muscle properties based on healthy subject data and does not account for CP-related changes in muscle–tendon unit parameters, which are known to influence muscle forces [32] and JRF estimates [33]. Second, subject-specific bone morphology was not modeled, despite its demonstrated impact on JRFs [34]. Neither patient-specific muscle contractile and architectural properties nor skeletal deformities could be practically incorporated into the simulated musculoskeletal model due to ethical and technical challenges in measuring these parameters. The primary reason for employing the Rajagopal model was the unavailability of a comprehensive CP-specific model that could accommodate the unique characteristics of our patient population. Developing such a model would require extensive subject-specific data, including detailed muscle–tendon unit properties and skeletal morphology, which were not feasible to obtain within the scope of this study. However, CP-related changes especially in muscle strength, stiffness, and activation patterns might alter the magnitude, timing, and orientation of the JRFs. For instance, altered muscle–tendon dynamics in CP could lead to increased joint loading due to elevated co-contraction of antagonistic muscles.

Third, this study did not account for the elevated co-contraction of antagonistic muscles observed in CP. In TD individuals, co-contraction can add up to 1 body weight (BW) of force to tibiofemoral contact forces [35]. In CP, this co-contraction is even more pronounced [36], likely contributing substantially to joint loading. This limitation arises from the absence of electromyography (EMG)-informed methods [37], which would have been necessary to capture these effects. Differences in muscle activation patterns in CP may alter the timing of peak forces or change the contribution of individual muscles to joint loading. Gharehbolagh et al. (2023) reported significantly greater muscle co-activation in individuals with CP compared to typically developing individuals, particularly for lower leg and thigh agonist/antagonist muscle pairs during walking [38]. Incorporating EMG into musculoskeletal modeling in future studies could provide a deeper understanding of how muscle activation patterns, including co-contraction, influence joint forces and gait efficiency in CP patients.

Another limitation of this study is the sample size. The relatively small sample size is attributed to the rarity of the specific population under investigation (children with CP exhibiting mild crouch gait) and the extensive data collection process involved. Despite this limitation, the study offers valuable insights into JRFs in this underrepresented population.

Future studies can incorporate subject-specific bone geometries obtained from imaging modalities such as MRI or CT scans. These geometries can be integrated into musculoskeletal models to better represent the anatomical variations in patients with CP, including femoral neck-shaft angles and torsional abnormalities. Subject-specific bone morphologies will also facilitate the accurate placement of muscle attachment points and adjustments to muscle–tendon unit parameters. This would improve the estimation of muscle forces and their contributions to JRFs. Combining 3D imaging data with gait analysis and EMG could provide a more comprehensive understanding of how bone morphology interacts with muscle dynamics and gait patterns. Future attempts with larger cohorts and multi-center collaborations are recommended to validate and generalize the findings.

## 5. Conclusions

We found that the major lower limb joint reaction forces in patients of CP with mild crouch gait differ from those of TD subjects in magnitude but not in direction and timing. The hip joint reaction forces in CP were lower than normal in their anteroposterior and vertical components, with the same for the anteroposterior component of the ankle JRF. Knee JRFs were elevated slightly above normal, as were the remaining components of other JRFs. Despite having several similarities with JRFs for CP reported in the literature using generic models, the impacts of variability in patient gait pattern and employed musculoskeletal model were major differences in the orientation of predicted mediolateral joint reaction forces and occasional differences in the timings and magnitudes of peak JRFs. The results of our study highlight the need for standardizing musculoskeletal simulation-based analyses in CP due to the prevalent variability in patient groups and model selection.

## Figures and Tables

**Table 1:** Average RMSD values representing the level of differences between the joint reaction forces obtained for patients with cerebral palsy and typically developing individuals.

		Hip Joint	Knee Joint	Ankle Joint
JRF component				
Anterior–posterior	Mean	0.15	0.24	0.26
	Max	0.19	0.28	0.29
	Min	0.12	0.22	0.24
Vertical	Mean	0.39	0.28	0.32
	Max	0.42	0.31	0.36
	Min	0.37	0.24	0.30
Mediolateral	Mean	0.41	0.22	0.27
	Max	0.45	0.24	0.32
	Min	0.39	0.19	0.24
	*p*-values			
Anterior–posterior vs. vertical		**0.012**	0.051	0.048
Anterior–posterior vs. mediolateral		**0.012**	0.055	0.046
Vertical vs. mediolateral		0.052	0.049	0.048

*p*: level of statistical significance between compared pairs. Max and Min represent the maximum and minimum RMSD values, respectively. Significant differences are highlighted in bold.

**Table 2:** Average PCC values quantifying how strongly and in which direction (positive or negative) the JRFs obtained from patients with cerebral palsy and typically developing individuals were related to each other.

		Hip Joint	Knee Joint	Ankle Joint
JRF components				
Anterior–posterior	Mean	0.91	0.90	0.81
	Max	0.92	0.92	0.82
	Min	0.89	0.89	0.79
Vertical	Mean	0.83	0.89	0.84
	Max	0.85	0.91	0.85
	Min	0.81	0.88	0.83
Mediolateral	Mean	0.84	0.87	0.79
	Max	0.85	0.88	0.81
	Min	0.82	0.86	0.77
	*p*-values			
Anterior–posterior vs. vertical		**0.013**	0.023	**0.014**
Anterior–posterior vs. mediolateral		**0.014**	**0.014**	0.017
Vertical vs. mediolateral		0.057	0.021	**0.014**

*p*: level of statistical significance between compared pairs. Max and Min represent the maximum and minimum PCC values, respectively. Significant differences are highlighted in bold.

## Data Availability

The data used in this study was made available publicly by [19].
